# Corrosion Resistance to Chloride of a Novel Stainless Steel: The Threshold Chloride Value and Effect of Surface State

**DOI:** 10.3390/ma12142235

**Published:** 2019-07-11

**Authors:** Hailong Wang, Yuanjian Wu, Xiaoyan Sun, Jiayan Ling, Daoqin Zou

**Affiliations:** Department of Civil Engineering, Zhejiang University, Hangzhou 310058, China

**Keywords:** Stainless steel, corrosion resistance, threshold chloride concentration, surface damage

## Abstract

To evaluate the corrosion resistance of a novel stainless steel intended for use within reinforced concrete (RC) structures exposed to aggressive environments, the threshold chloride concentration of three stainless steels (316, 2205, novel 2205) and two carbon steels (HRB400, HRB500) exposed to pore solutions of fresh concrete was experimentally studied by means of electrochemical methods. The effect of steel surface state on the corrosion resistance was also experimentally investigated. The results showed that the novel stainless steel has a much higher corrosion resistance than those of the carbon steels and stainless steels when subjected to chloride environments. The presence of surface damage leads to significant decrease of corrosion resistance for carbon steel, however the corrosion can be certainly inhibited with the accumulation of rust on the steel surface. Although the oxide layer was worn, the novel 2205 stainless steel still has a great corrosion resistance.

## 1. Introduction

Reinforced concrete (RC) structures are one of the most affordable structures used globally. However, conventional RC structures have poor durability, especially under severe corrosion conditions, such as ocean, de-icing, or other chloride-contaminated environments. A protective, thin oxide layer forms on the surface of steel when it is exposed to a highly alkaline medium, however this layer can be destroyed when it is exposed to a certain concentration of chloride ions [1]. Considering the issues associated with corrosion, the use of stainless-steel reinforcement appears to be the most appropriate solution to increase the durability of RC structures existing in aggressive environments [2,3,4,5,6]. The use of stainless steels can essentially improve the corrosion resistance of embedded bars; this is because the alloying elements of chromium, nickel, and molybdenum can result in the formation of a more stable oxide layer, which protects the metallic matrix beneath the film from chloride corrosion [7,8]. However, the use of stainless steels increases the cost of the construction phase, and thus their use is limited [9]. Nevertheless, based on the results of life-cycle cost analysis, the use of stainless steel is considered to be a more economical alternative. 

Stainless steel is passive; however, it is also susceptible to localized corrosion in the presence of chloride ions [10]. For corrosion evaluation, its exact value of threshold chloride concentration for pitting initiation is a very important factor. Typical electrochemical techniques, such as electrochemical impedance spectroscopy (EIS) and the linear polarization technique (LPR), were often used to define the critical chloride content. In addition, Cabrini et al used cyclic voltammetry (CV) tests to study the effect of chloride content on corrosion initiation, and then assessed the effect of inhibitor addition on the critical chloride content [11]. Consequently, many studies had been carried out to determine the corrosion behaviorbehavior of stainless steel bars that were embedded in concrete or submerged in simulated pore solutions [12,13,14,15,16,17,18]. Freire et al. focused on the corrosion behaviorbehaviors of AISI304 and AISI 316 stainless steels that were exposed to simulated pore solutions with various pH levels and chloride concentrations [10,19]. The results of their studies showed that the pH of a solution is important with regard to the evolution of the resistance of the film and the charge-transfer process. Bertolini et al. studied the influence of carbonation on the critical chloride concentration [20]. They found that the value of the critical chloride concentration decreased as the testing temperature increased. However, contrary results were obtained when solutions with high carbonate/bicarbonate concentrations were used, where the corrosion appeared to be inhibited. Moser et al. examined the chloride induced corrosion resistance of austenitic, duplex, and martensitic high-strength stainless steels in simulated alkaline and carbonated concrete solutions [21]. They found that in alkaline solutions, all high-strength stainless steels showed high corrosion resistance, however, when exposed to carbonated solutions, corrosion resistance was reduced. Furthermore, Kouřil et al. determined the relationship between the superficial state of the steel and the corrosion resistance; they confirmed that steels with scaled surfaces had lower resistance, even in the cases of stainless steels with high alloying contents [22]. 

As discussed above, the majority of the studies focused on the corrosion resistance of various stainless steels; however, few studies considered the exact value of the threshold chloride concentration for pitting initiation. Both for the design of new structures and for condition assessment of existing structures, the value of threshold chloride concentration is important as the remaining service life is often considered as the time required to reach the chloride threshold value at the depth of the reinforcement [23,24,25]. Therefore, the primary objective of this study is to determine the critical chloride values of steels for their appropriate application. To accomplish this, three stainless steels (type-316 austenitic steel, type-2205 duplex steel and a novel type-2205 duplex steel) and two carbon steels (HRB400, HRB500) were electrochemically characterized in simulated pore solutions. 

The corrosion resistance depends highly on the passive layer of the steel. This layer was often damaged in the application, which may contribute to the pitting corrosion of the steel that was reported by Cabrini [26,27]. However, few studies have discussed the effect of surface state on the stainless steel resistance. Therefore, another goal of this study is to clarify the effect of surface state on the corrosion resistance of stainless steel.

## 2. Experimental Program

### 2.1. Materials, Specimens and Solutions

**Figure 1:**
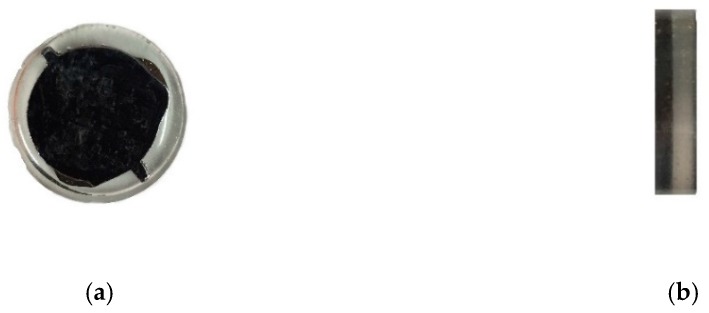
Specimen: (**a**) Top view; (**b**) side view.

A type-2205 duplex stainless steel, a novel type-2205 duplex stainless steel, a type-316 austenitic stainless steel, and two types of carbon steels (HRB400, HRB500) were tested in this study. The main chemical components of these steels were determined via energy dispersive X-ray spectrometry (Quanta 650 FEG, FEI, Hillsboro, Oregon, USA), as shown in Table 1. Specimens were cut from the steel bars, and were manufactured into cylinders with a diameter of 12 mm and height of 1.5 mm. As shown in Figure 1, the sides and one cross section of each specimen were covered with epoxy, and the other cross section was polished, which was considered as the working electrode. The specimen was cleaned with deionized water, and then the surface was wiped with anhydrous ethanol.

A concrete pore solution with a pH value of 12.5 was prepared to simulate the electrolyte contained within the pores of fresh concrete. This alkaline solution was prepared by diluting 0.01 M Ca(OH)_2_ and 0.03 M NaOH with deionized water. To avoid carbonation, the solution was used immediately, and its pH was measured and maintained using pH meters. The specimens were submerged in the solutions for about 5 days (120 h), which ensured the complete formation of the passive film [28]. Subsequently, the carbon steels and stainless steels were immersed successively into the simulated pore solutions with chloride ions of 0.01–0.5 M. At a certain concentration, potentiodynamic polarization curve and impedance spectroscopy were tested after 1d stabilization. After the tests, the specimens were immersed into a simulated solution with higher chloride ion concentration, and so on.

### 2.2. Surface Treatment

**Figure 2:**
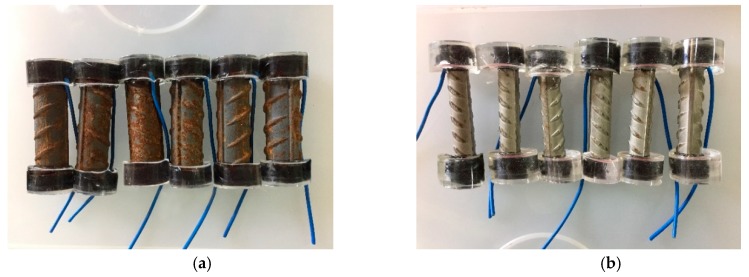
Specimens: (**a**) HRB500; (**b**) novel type-2205 duplex stainless steel.

The novel type-2205 duplex stainless steel and the carbon steel HRB500 were used to figure out the effect of surface damage on the corrosion behavior of steel. In order to obtain two different surface states, 6 specimens were prepared for each steel and half of them were filed to simulate the worn damage of oxide layer. Specimens with 60 mm length were cut from steel bars, the sides of each specimen were covered with epoxy as shown in Figure 2. The middle part with 40 mm length was left as working electrode, neglecting the influence of steel rib on the electrode area. 

A concrete pore solution with a pH value of 9 was prepared to simulate the electrolyte contained within the pores of carbonated concrete. This alkaline solution was prepared by diluting 0.015 M NaHCO_3_ and 0.005 M Na_2_CO_3_ with deionized water. To accelerate the corrosion, chloride ions of 0.6 M was added into the solution. After 3 months of a dry-wet cycle (wet 3d, dry 4d), the chloride ions in the solution were increased to 0.85 M.

### 2.3. Electrochemical Tests

An electrochemical test system, Reference 600 Potentiostat (Gamry instruments, Philadelphia, PA, USA), was used to perform the electrochemical experiments at room temperature, ~25 °C. A three-electrode system was set up for an electrochemical test, in which the steel specimen was used as working electrode, platinum electrode was adopted as an assistant electrode, and mercury-mercury oxide electrode (Hg/HgO) was acted as reference electrode. The potential of the working electrode was determined based on that of the saturated calomel electrodes (SCE). 

Electrochemical impedance spectroscopy (EIS) and potentiodynamic polarization tests were carried out to measure the corrosion states of the steels. Both measurements commenced following the stabilization of the open-circuit potential (OCP). During the EIS measurement, the frequency was swept from 10^6^ Hz to 10^−3^ Hz, with an applied alternating current (AC) amplitude of 10 mV. The charge-transfer resistance (*R*_ct_) was obtained through Zview 3.2c software (Gamry instruments, Philadelphia, PA, USA) via experimental data fitting. 

Potentiodynamic curves were carried out starting at −70 mV (vs OCP) to + 70 mV (vs OCP) at 0.167 mV/sec scan rate. The corrosion potential (*E*_corr_), corrosion current density (*i*_corr_), polarization resistance (*R*_p_), and corrosion rate (*CR*) were fitted from the polarization curves, using the Gamry Echem Analyst software. In the case of unintended crevice corrosion, the test was considered to be invalid and repeated tests were carried out using new specimens.

Based on the electrochemical signal values obtained as the chloride-ion concentration was increased, the critical chloride concentration was determined for each steel. 

## 3. Results and discussion

### 3.1. Polarization Curves of the Steels Exposed to Pore Solutions with Chloride Ions

**Figure 3:**
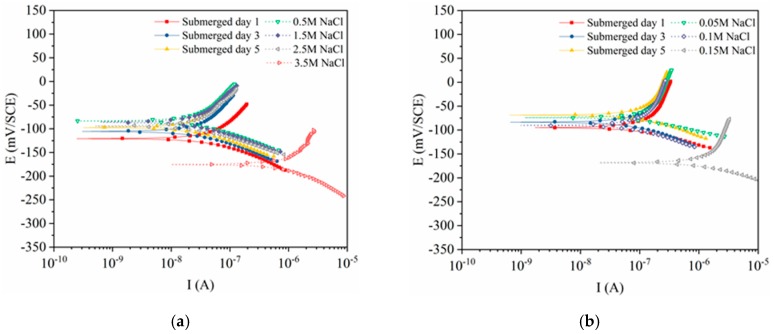

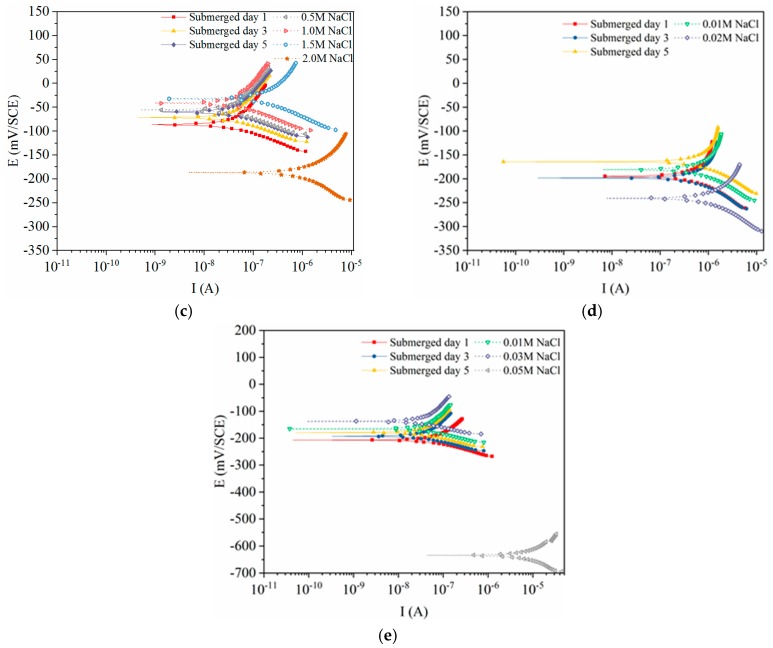
Potentiodynamic polarization curves obtained for the steels: (**a**) novel type-2205 duplex stainless steel; (**b**) type-2205 duplex stainless steel; (**c**) type-316 austenitic stainless steel; (**d**) HRB400; (**e**) HRB500.

Figure 3 shows the polarization curves obtained for the five types of steels. All of the curves indicate that the corrosion potential will increase during the passivation period, and will tend to stabilize over time. When the chloride ions were initially added, the novel type-2205 and type-2205 duplex stainless steel, type-316 austenitic stainless steel remained stable; however, the two carbon steels were slightly affected. Although HRB400 and HRB500 belong to a same type of steel, their chemical compositions are slightly different. As a result, the corrosion potential of HRB500 was much lower than that of HRB400 when corrosion happened. With regard to the shapes of the curves, no changes can be observed prior to the occurrence of the critical pitting corrosion. As shown in the figures, the curves shifted downwards and towards the right when the chloride concentration reached the critical value. 

### 3.2. EIS Curves of the Steels Exposed to the Pore Solutions with Chloride Ions

**Figure 4:**
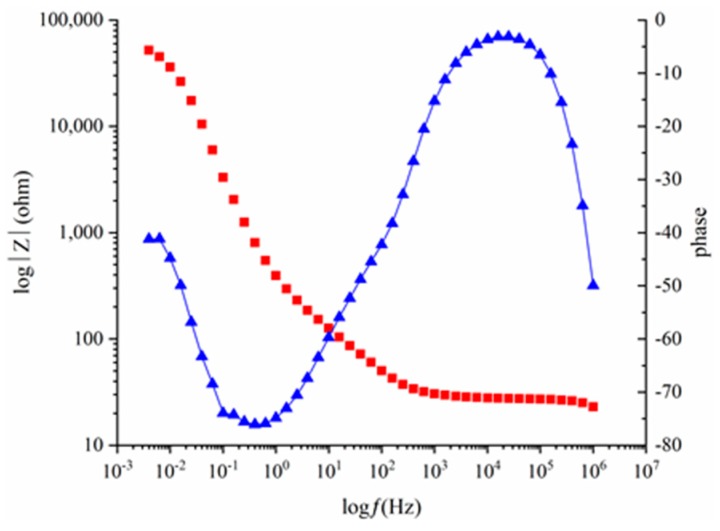
Bode curves of type-2205 duplex stainless steel in 0.5 M NaCl solution.

**Figure 5:**
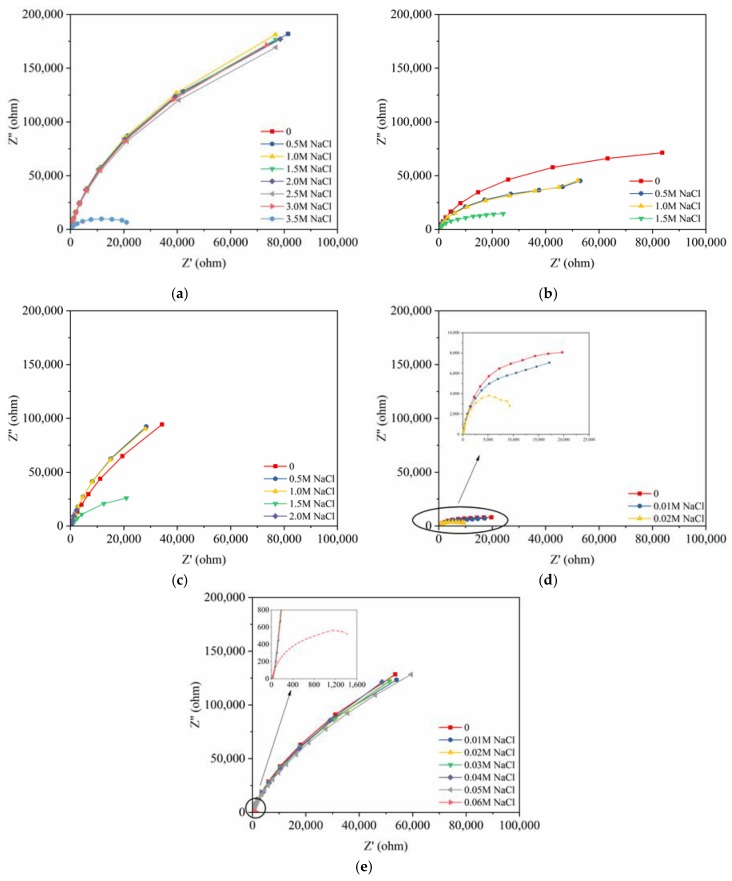
Nyquist curves of the steels based on the EIS measurements: (**a**) novel type-2205 duplex stainless steel; (**b**) type-2205 duplex stainless steel (**c**) type-316 austenitic stainless steel; (**d**) HRB400; (**e**) HRB500.

Figure 4 shows the Bode curve of 2205 duplex stainless steel in 0.5 M NaCl solution. It is clear that there are two time constants. The Nyquist curves, based on the results of the EIS tests, are displayed in Figure 5. The radii of the semicircles were related to the charge-transfer resistance of the passive film of the steels; as the resistance increased, the radius of each semicircle increased. As the chloride-ion content increased, the radii of the semicircles initially slightly decreased, and then significantly decreased, indicating the destruction of the passive film. Curves indicated that the novel type-2205 duplex stainless steel had higher charge-transfer resistance of the passive film with the simulated concrete pore solution.

### 3.3. Electrochemical Characteristics and Threshold Chloride Concentrations

*E*_corr_, *I*_corr_ and the polarization resistance (*R*_p_) values can be obtained from the potential polarization curve, a potential range of 5 mV above and below the corrosion potential was used to fit *R*_p_. Moreover, the Corrosion Rate (*CR)* can be calculated using equation (1), where *M* is the Molar mass of iron (g/mol), *t* is the time of a year (s), *z* = 2 is the valence of iron, *F* is the Faraday constant, *d* is the density (g/m^3^), and *A* is area of the specimen (m^2^)

(1) $$CR=\frac{I_{\mathrm{corr}}\cdot M\cdot t}{zFdA}$$

Theoretically, the corrosion current density (*i*_corr_, defined as *I*_corr_/*A*), polarization resistance (*R*_p_), and *CR* are stable when the surface of the steel is passivated. Even when chloride ions are added to the solution, excessive fluctuation of these values will not occur. However, when the chloride ions accumulate to a certain extent, and attain the critical concentration that the steel can withstand, damage of the passive film will occur. Then, *i*_corr_ and *CR* will suddenly increase, and *R_p_* will suddenly decrease.

**Figure 6:**
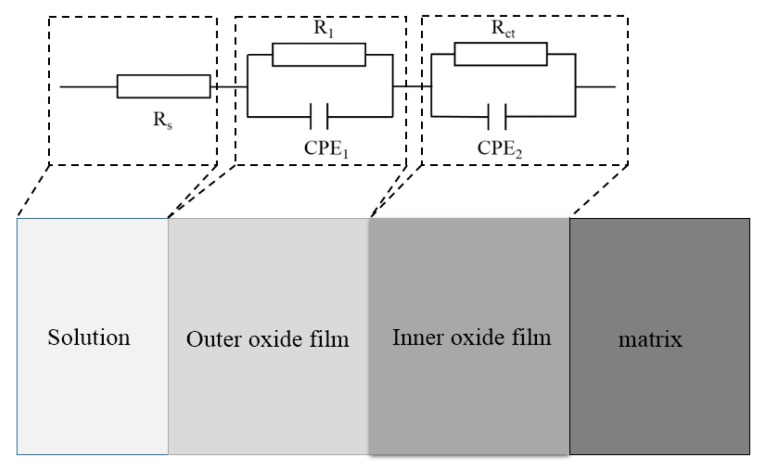
Equivalent circuit for an EIS fitting.

Blanco used a two-time constant model like Figure 6 to fit the obtained experimental EIS data of stainless steel in simulated pore solutions [21]. This type of circuit can be regarded as an electrical representation of a two-layer model of the oxide film, consisting of a barrier compact inner film and a porous outer layer [29]. This two-layer structure had also been hypothesized for the passive film on stainless steels [30]. According to above studies, both capacitors in the figure were modeled as constant phase elements (CPE), which represented the double layer formed on the surface of the material. Each circuit component has a different meaning. *R_s_* represents the ohmic resistance of the electrolyte at high frequencies. The medium-frequency time constants (*R*_1_ and CPE_1_) appear to be associated with a redox reaction that occurs on the surface of the steel. At low frequencies, the time constants (*R_ct_* and CPE_2_) represent the charge-transfer process. The polarization resistance (*R_p_*), based on the potentiodynamic polarization curve, has a similar meaning to that of *R_ct_*; these can both represent the state of the passive film. Furthermore, the *R_ct_* value, corresponding to the corrosion intensity, is similar to the *i*_corr_ value, obtained from the polarization test [23]. As the chloride-ion content increases, the passive film becomes unstable, and the charge-transfer resistance will decrease. Therefore, the destruction of the passive film can also be characterized by a sudden change in the charge-transfer resistance; the critical chloride concentration range can also be determined.

**Figure 7:**
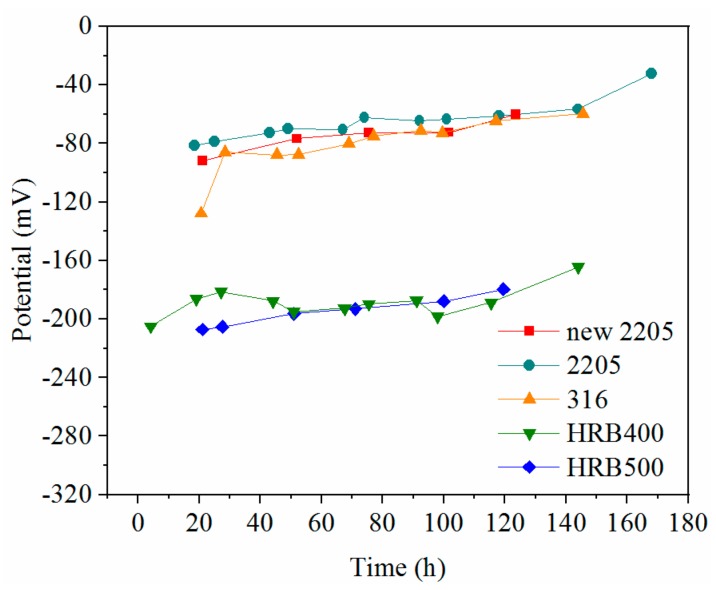
Natural corrosion potentials as a function of time.

Figure 7 presents the open circuit potentials as a function of the immersion period. The potentials of the three stainless steels were stabilized at −80 mV, and the potentials of the two carbon steels were stabilized at around −190 mV. 

**Figure 8:**
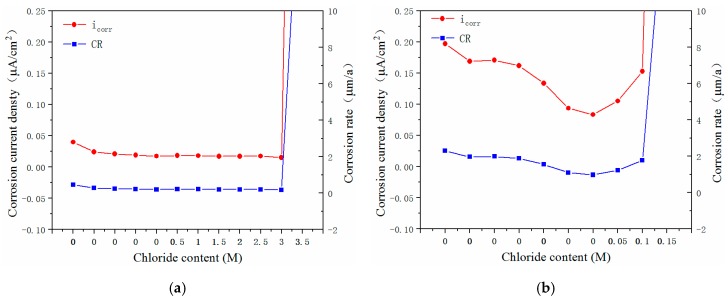

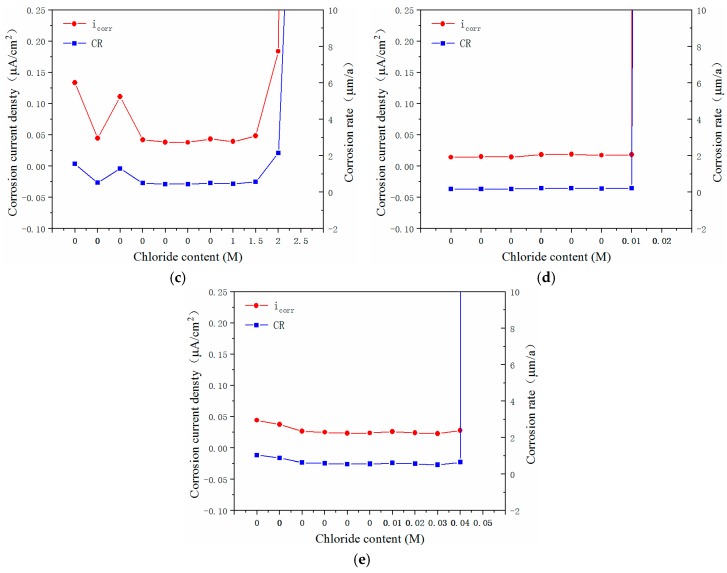
Influence of chloride content on the icorr and CR values of the steels: (**a**) novel type-2205 duplex stainless steel; (**b**) type-2205 duplex stainless steel (**c**) type-316 austenitic stainless steel; (**d**) HRB400; (**e**) HRB500.

**Figure 9:**
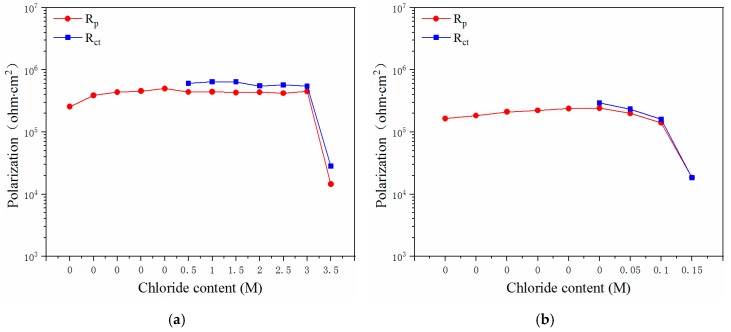

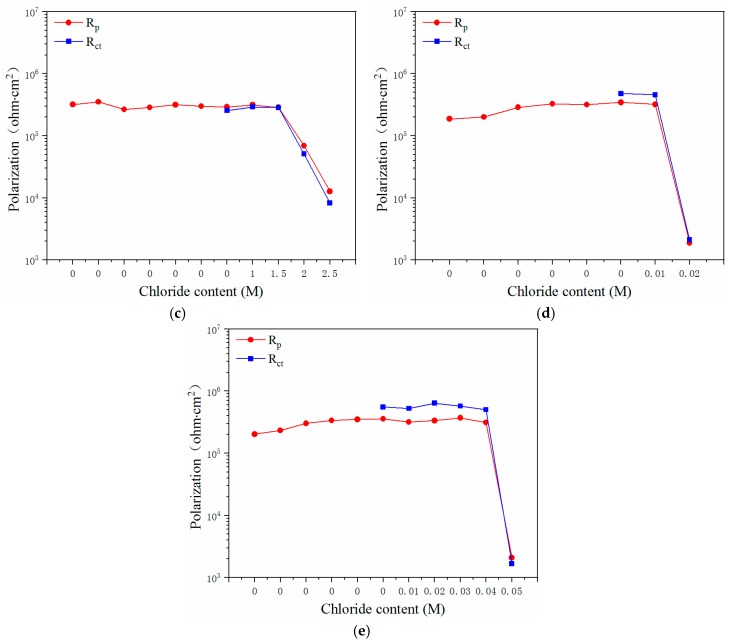
Influence of chloride content on the *R_p_* and *R_ct_* values of the steels: (**a**) novel type-2205 duplex stainless steel; (**b**) type-2205 duplex stainless steel (**c**) type-316 austenitic stainless steel; (**d**) HRB400; (**e**) HRB500.

The corrosion current density (*i*_corr_), *CR*, polarization resistance (*R_p_*), and charge-transfer resistance (*R_ct_*) of the five types of steels that were immersed in the concrete pore solution were obtained, and Figure 8 and Figure 9 present the fitting results obtained for four electrochemical parameters as a function of the chloride content.

Figure 8a shows the *i*_corr_ and *CR* values obtained for the novel type-2205 duplex stainless steel that was exposed to the solutions with various chloride concentrations, and Figure 9a shows the respective *R_p_* and *R_ct_* values. As shown, following immersion, the *i*_corr_ and *CR* values decreased slightly and then remained stable. When the corrosion process was initiated, there was a hundredfold rise in the *i*_corr_ and *CR* values; a similar phenomenon occurred when the chloride content of the solution increased from 3.0 M to 3.5 M. Simultaneously, the *R_p_* and *R_ct_* values decreased 31-fold and 19-fold, respectively. It can be concluded that in the case of the novel type-2205 duplex stainless steel, the critical chloride concentration, at a pH of 12.5, was in the range of 3.0–3.5 M. Furthermore, it can be determined that in the cases of the type-2205 duplex, type-316 austenitic, HRB400, and HRB500 steels, the critical chloride concentrations were 0.1–0.15 M, 1.5–2.0 M, 0.01–0.02 M, and 0.04–0.05 M, respectively, as summarized in Table 2. 

Amongst the various steels, the novel type-2205 duplex stainless steel exhibited the best resistance to chloride corrosion; its critical chloride concentration was at least 20 times greater than that of the type-2205 duplex stainless steel, and more than 1.5 times greater than the austenitic 316 stainless steel. As for those of the carbon steels, the critical chloride concentration of the novel type-2205 duplex stainless steel was 2 orders of magnitude higher than them.

When the chloride concentration of the solution did not reach the critical threshold, the *i*_corr_ and *CR* values of the surface of the specimen remained stable; these values were mostly below 0.05 μA/cm^2^ and 1.2 μm/a, respectively. In addition, the *R_p_* and *R_ct_* values of the four steels were all within the range of 10^5^–10^6^ ohm.cm^2^. This indicates that the corrosion states of the surfaces of the steels are identical to those prior to the occurrence of corrosion. 

However, following the occurrence of corrosion, the *i*_corr_ and *CR* values of the three stainless steels increased by two orders of magnitude, while the *i*_corr_ and *CR* values of the carbon steels increased by three and four orders of magnitude, respectively, indicating that more severe corrosion occurred in the case of the carbon steels, where pitting corrosion occurred. Moreover, the *R*_p_ and *R*_ct_ values of the novel duplex stainless steel reduced by one order of magnitude, while those of the other three steels all decreased by two orders of magnitude.

The stainless steels exhibited superior corrosion behaviors to those of the carbon steels, and therefore could be employed in RC structures exposed to chloride-containing environments. Moreover, comparing the electrochemical characteristics of the four steels, it can also be concluded that the novel type-2205 duplex stainless steel possesses the highest corrosion resistance. Considering the compositions of the steels, compared with type-2205 duplex stainless steel and type-316 austenitic stainless steel, the novel type-2205 duplex stainless steel had higher chromium and molybdenum content, and consequently offered the greater corrosion resistance. Nickel is an important element in austenitic stainless steel with high cost. However, it plays little role in pitting resistance equivalent number (PREN) for stainless steel. Meanwhile, this novel steel is cost effective because of its low nickel content, which consequently promotes the use of such stainless steels in concrete structures.

### 3.4. Effect of Surface State on Corrosion Characteristics

**Figure 10:**
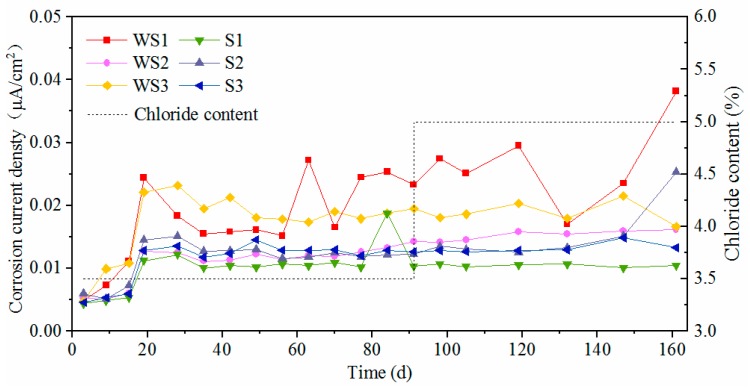
Corrosion current density of novel 2205 duplex stainless steel.

**Figure 11:**
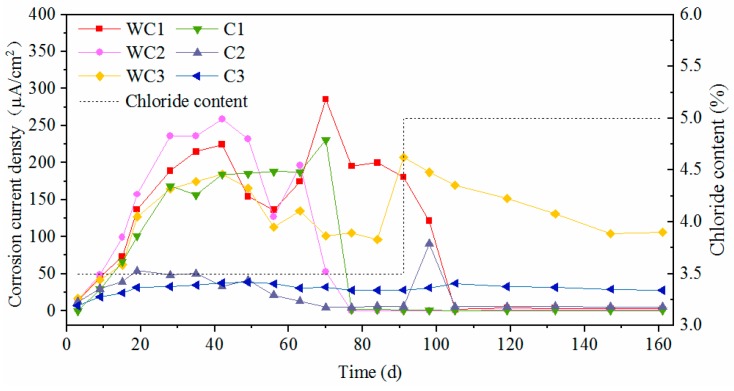
Corrosion current density of HRB500 carbon steel.

**Figure 12:**
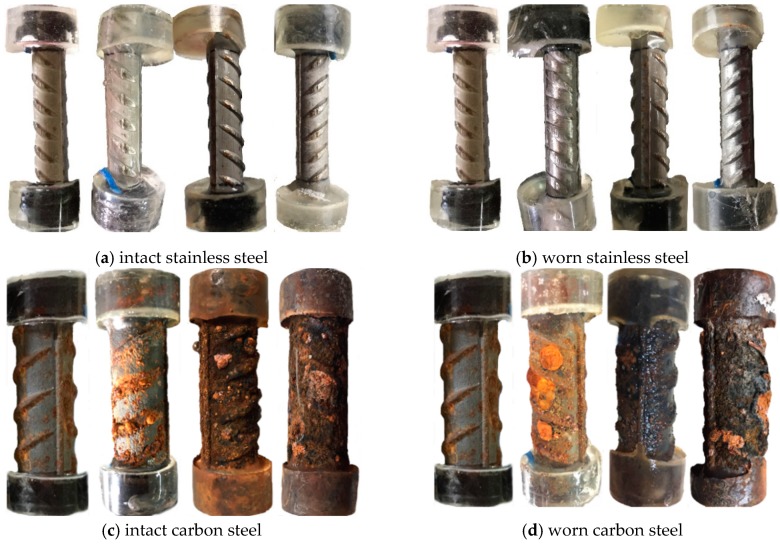
Corrosion state of intact and worn stainless and carbon steels after 0, 10, 90 and 160 days’ exposure.

Figure 10 and Figure 11 show the time-varying corrosion current density of the test steels, in which WS and S represent the worn and intact stainless steel, WC and C represent the worn and intact carbon steel respectively, and 1–3 represents the three parallel specimens of a steel. The corrosion current density of novel 2205 duplex stainless steel was almost within the range of 0.01–0.03 μA/cm^2^, that was much lower than the critical current density of corrosion [31]. At the initial stage, the corrosion current density increased continually and tended to stabilize after 20 days. The worn stainless steel specimens exhibited higher corrosion current density as compared to the intact specimens, but the current densities still did not reach the critical value of corrosion, which means this type of stainless steel has a great corrosion resistance even though its oxide layer was damaged. Furthermore, the increasing chloride concentration had few effects on the corrosion rate of stainless steel. Both WS and S maintained a very low corrosion rate, and it was hard to find any change on the surface of stainless steel after 160d exposure, as shown in Figure 12.

As for carbon steels, the corrosion current densities exceeded 0.2 μA/cm^2^ initially and then increased severely until 40d. As the passive film of carbon steel was difficult to form in the carbonated solution, the corrosion happened promptly after immersing the steel into the chloride solution. After that, the values fluctuated violently in the duration of 40 d to 90 d and then decreased gradually to stable values. Since the rust can separate the steel substrate from chloride and oxygen, this phenomenon can be explained by the accumulating rust on the steel surface. The corrosion current density for worn carbon steel was almost within the range of 100–250 μA/cm^2^ most of the time, however it was within 0.1–2 μA/cm^2^ for the intact specimen. Obviously, the presence of surface damage leads to a significant decrease on the corrosion resistance of carbon steel.

**Figure 13:**
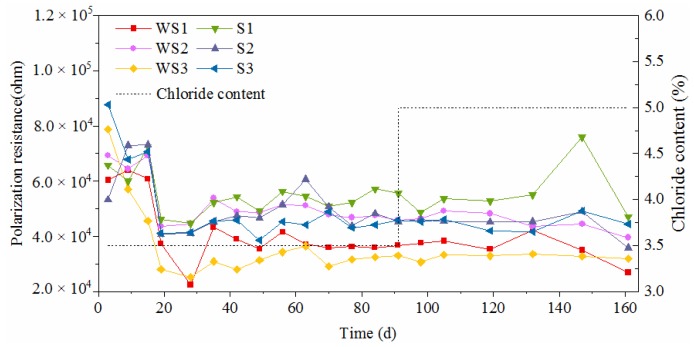
Polarization resistance of novel type-2205 duplex stainless steel.

**Figure 14:**
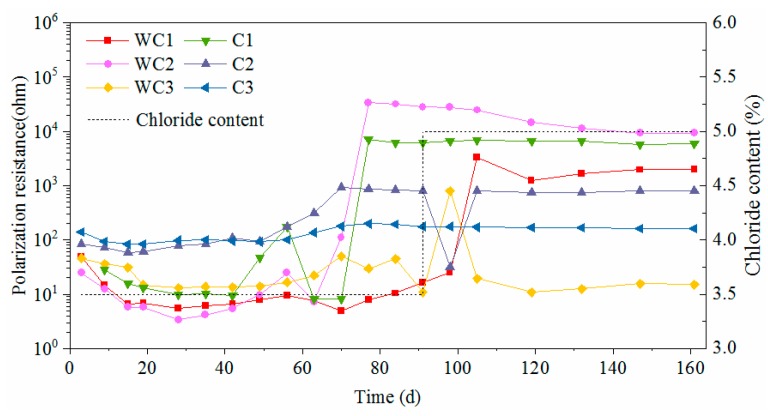
Polarization resistance of HRB500 carbon steel.

Figure 13 and Figure 14 display the time-varying polarization resistances of the test steels. They also indicate that the presence of surface damage declines the polarization resistance of novel 2205 and HRB500 steels. The polarization resistances of intact stainless specimens in the solution with a PH value of 9 kept in the range of 2–6 × 10^4^ ohm, which was an order of magnitude lower than that in the solution with a PH value of 12.5. Although the solution alkalinity was reduced by the carbonization, the passive film of stainless steel still could be formed, however the quality was not as high. For carbon steel, the passive film did not form in the carbonated solution. Therefore, the polarization resistances of worn carbon steels lied in the range of 20–50 ohm at the initial stage. Even for intact specimens, the polarization resistances of carbon steels were no more than 200 ohm. As shown in Figure 12, rust accumulated continuously on the surface of carbon steel, which effectively inhibited the ferric ions from contacting the chloride and the oxygen. As a result, the polarization resistances of some carbon steel specimens increased to 10^3^–10^4^ ohm due to the rust effect after around 3 months of dry-wet cycles.

## 4. Conclusions

The critical chloride concentrations of five different steel bars and the effect of surface state on corrosion resistance were experimentally investigated in this study. Based on the test results, the following conclusions can be drawn:

- The potentials of the stainless steels were 110 mV greater than those of the carbon steels, indicating that they were less likely to corrode when exposed to a chloride-containing environment.
- The critical chloride concentration of the novel type-2205 duplex stainless steel was at least 20 times greater than that of the type-2205 duplex stainless steel, 1.5 times greater than that of the type-316 austenitic stainless steel, and was significantly greater than that of the carbon steels. This indicated that, compared with the other steels, the novel type-2205 duplex stainless steel offered superior resistance to chloride ions and could remain passivated for a longer period of time.
- The element content of the steel bar had a significant influence on its corrosion resistance. Higher chromium and molybdenum content in this novel type-2205 duplex stainless steel offered greater corrosion resistance. Simultaneously, the low nickel content reduced the initial cost associated with its use.
- The presence of surface damage led to significant decrease of corrosion resistance for carbon steel. Although the oxide layer was destroyed, the novel type-2205 stainless steel still showed a great corrosion resistance.
- The corrosion can be inhibited to a certain degree with the accumulation of rust on the steel surface for carbon steel. However, the rust cannot be accumulated on the surface of novel type-2205 duplex stainless steel in a short period of time, due to its low corrosion rate.

## Figures and Tables

**Table 1:** Chemical compositions of the steels (% wt).

Composition	C	Mn	S	Si	Cr	Ni	Mo
HRB400	0.23	1.58	–	0.32	1.35	–	–
HRB500	0.25	1.62	0.06	0.12	0.31	–	–
316	0.03	0.93	0.03	0.24	16.56	11.07	2.23
2205	0.03	0.82	0.01	0.23	20.95	5.84	2.57
Novel 2205	0.02	1.36	–	0.14	22.83	6.15	3.21

**Table 2:** Critical chloride concentrations of steels.

Type of Steel	New 2205	2205	316	HRB400	HRB500
Critical value	3.0–3.5 M	0.1–0.15 M	1.5–2.0 M	0.01–0.02 M	0.04–0.05 M
